# Integrated Multi-Omics and Machine Learning Framework Identifies Diagnostic Signatures and Druggable Targets in Breast Cancer

**DOI:** 10.3390/genes17040396

**Published:** 2026-03-30

**Authors:** Zifu Wang, Jinqi Hou, Yimin Chen, Jundi Li, Sivakumar Vengusamy

**Affiliations:** 1 School of Computing, Asia Pacific University of Technology & Innovation, Kuala Lumpur 57000, Wilayah Persekutuan Kuala Lumpur, Malaysia; tp089377@mail.apu.edu.my (Z.W.); 15989437470@163.com (Y.C.); 2 Department of Biological Sciences, Mellon College of Science, Carnegie Mellon University, 4400 Fifth Ave, Pittsburgh, PA 15213, USA; jinqih@andrew.cmu.edu; 3 Beijing Institute of Remote Sensing Equipment, China Aerospace Science and Industry Corporation Limited, Beijing 100854, China; lijundi-a@163.com

**Keywords:** breast cancer, multi-omics integration, machine learning, CHEK1, drug repurposing

## Abstract

Background: Breast cancer (BC) is one of the most diagnosed malignancies and a leading cause of cancer-related mortality among women worldwide, thereby posing a substantial threat to women’s health worldwide. However, clinically robust diagnostic biomarkers with high sensitivity and specificity, as well as well-validated molecular targets for targeted therapy, remain limited. Methods: BC transcriptomic data from seven GEO datasets and the TCGA-BRCA cohort (*n* = 1231) were integrated for analysis. After batch-effect correction, candidate genes were screened through DEA, WGCNA, and PPI networks analysis. An ensemble machine learning (ML) framework incorporating 127 algorithmic combinations was constructed, and SHAP analysis was applied to identify hub genes. Further analyses included functional enrichment, immune infiltration, miRNA regulatory network analysis, and SMR analysis. The expression patterns were validated using single-cell transcriptome data. Drug repositioning analysis and AI-assisted virtual screening were performed to prioritize compounds with favorable drug-like properties. The predicted binding modes of candidate compounds with CHEK1 were assessed by molecular docking. Results: Thirty core genes were obtained through differential expression, WGCNA, and PPI screening. Integrated ML (127 algorithms) determined the optimal model (AUC = 0.919), and SHAP identified nine feature genes, among which CHEK1 and KIF23 showed preliminary diagnostic potential across four external cohorts (AUC: 0.625–0.938). Functional enrichment indicated that both are enriched in the cell cycle and p53 pathways, closely associated with BRCA1/ATR; immune infiltration revealed significant correlations with macrophages and CD8^+^ T cells, with hsa-miR-15a-5p and hsa-miR-607 being common upstream regulatory miRNAs. SMR analysis supported a causal relationship between CHEK1 expression and BC genetic susceptibility (*p*_SMR < 0.05, *p*_HEIDI > 0.05); single-cell analysis confirms its heterogeneous expression. AI-assisted virtual screening identified 25 A-grade computational candidate compounds from 171 candidates. Molecular docking suggested that Olaparib and LY294002 can form favorable interactions with the CHEK1 active pocket. Conclusions: The study identified CHEK1 as a key diagnostic gene for BC through 127 ML algorithms and SMR causal inference. By combining AI-assisted virtual screening and molecular docking, computational candidate compounds targeting CHEK1 were prioritized. These findings represent hypothesis-generating in silico predictions and require experimental validation before any therapeutic conclusions can be drawn.

## 1. Introduction

Breast cancer (BC) is a heterogeneous disease and frequently spreads to the bone and brain. In 2022, approximately 2.3 million new cases of BC were diagnosed globally, with about 666,000 deaths, ranking it as the leading malignant tumor among women [1] and one of the primary causes of cancer mortality in females [2,3,4,5]. Research indicates that any woman has a risk of developing BC, and its incidence increases with age, with the lifetime risk of BC in developed countries reaching as high as 12–13% [6]. Although targeted therapy and immunotherapy have made remarkable progress recently, the incidence rate of BC continues to rise [7]. This trend points to the need to improve early diagnostic strategies and treatment approaches. Due to the frequent lack of specific symptoms in early BC, some patients are already at an advanced stage at initial diagnosis, leading to increased treatment difficulty and poor prognosis [8]. Early and accurate diagnosis is key to reducing BC mortality and improving patient prognosis, while biomarkers with high sensitivity and specificity are important for early screening, auxiliary diagnosis, treatment monitoring, and prognostic evaluation [9]. The best biomarkers will be involved in several tumor-related processes and can be used in clinical settings to help create personalized treatment plans.

Early identification and accurate diagnosis of BC are highly complex clinical challenges, while artificial intelligence technology is gradually transforming the tumor diagnosis and treatment model, assisting clinical decision-making and improving patient prognosis [10]. Multiple studies have attempted to develop machine learning (ML) models for predicting BC risk and early diagnosis [11,12], among which algorithms such as random forest and support vector machine are considered effective tools for screening key tumor genes and constructing diagnostic models [13]. The evaluation of rapidly measured gene expression characteristics beyond traditional pathological indicators will provide clinicians with diagnostic and prognostic value [14]. However, the molecular mechanisms involved in the occurrence and development of BC remain far from elucidated. Currently, no consensus has yet been reached regarding gene expression-based biomarkers that can be directly used for clinical diagnosis of BC. Therefore, identifying reliable gene biomarkers for BC diagnosis has important clinical value.

ML plays an increasingly important role in analyzing gene expression data. Gene expression data usually has characteristics such as high dimensionality, high noise, and small sample size, containing a large amount of complex information [15]. ML algorithms, such as random forest, support vector machine, neural network, and deep learning, have been widely applied in the analysis and mining of gene expression data. By combining bioinformatics analysis with various ML algorithms, core genes closely related to tumor occurrence and development can be effectively identified, providing important references for early tumor warning, molecular diagnosis, and targeted therapy [16,17]. However, research using systematic multi-algorithm integration strategies—encompassing 127 feature-selection–classifier combinations together with genetic support and AI-based drug candidate prioritization—to identify and characterize BC diagnostic biomarkers remains limited. The innovation of the present work lies primarily in its integrative scope and multi-layer validation strategy, rather than in the development of a novel standalone algorithm.

In this study, we developed an integrated multi-omics and machine learning framework that systematically combines WGCNA, PPI network analysis, and 127 ML algorithms—substantially exceeding the scope of previous single-algorithm or dual-method approaches—coupled with SHAP interpretability, SMR causal inference, and AI-assisted virtual screening, to identify diagnostic biomarkers and druggable targets in BC. This multi-layered framework is designed to improve generalizability and reduce overfitting compared to prior studies relying on limited algorithm sets.

## 2. Methods

### 2.1. Study Design

**Figure 1:**
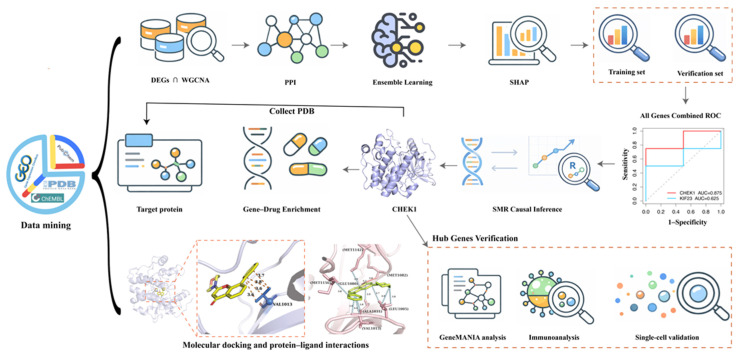
Study design flowchart.

This study integrated seven BC gene expression datasets from the GEO database and the TCGA cohort. After batch effect correction, differential expression analysis, WGCNA, and PPI network analysis, 30 candidate genes were identified. A total of 127 ML models were constructed to determine the optimal classifier, and key feature genes were selected using the SHAP algorithm. Subsequent analyses included functional enrichment, immune infiltration, miRNA regulation, SMR causal inference, single-cell transcriptome validation, and molecular docking. The workflow of this study is shown in Figure 1.

### 2.2. Data Collection and Analysis

A total of seven BC-related gene expression datasets (GSE100534, GSE162228, GSE89216, GSE74968, GSE99394, GSE98691, and GSE186344) were retrieved from the GEO database. Dataset inclusion criteria were as follows: (a) gene expression profiling by array or RNA sequencing; (b) inclusion of both tumor and matched normal breast tissue samples; (c) availability of raw or processed expression matrices; (d) public accessibility with adequate annotation; and (e) suitability for model training, external validation, or single-cell validation. GSE186344 was specifically selected for single-cell transcriptomic validation [18]. Batch effects across datasets were corrected using the sva package (v3.58.0), and principal component analysis (PCA) was performed to assess correction efficiency [19]. GSE100534 (normal = 16; tumor = 19) and GSE162228 (normal = 23; tumor = 110) were combined to form the training set, whereas GSE89216 (normal = 4; tumor = 4), GSE74968 (normal = 4; tumor = 4), GSE99394 (normal = 5; tumor = 5), and GSE98691 (normal = 4; tumor = 4) were merged as the external validation set and GSE186344 (normal = 3; tumor = 3) was used for the single-cell transcriptomic validation set. Additionally, omics data from 1231 female patients from the TCGA BC cohort were incorporated and processed with the same quality control and normalization procedures [20]. All expression matrices were log2-transformed and normalized using limma (version 3.66.0) normalizeBetweenArrays [21].

### 2.3. Differentially Expressed Genes (DEGs) Between the Training Set and TCGA Dataset

In the training set constructed by integrating GSE100534 and GSE162228, the screening threshold for DEGs was FDR < 0.05 and |log_2_FC| > 0.585; while in the TCGA dataset, to more rigorously screen for high-confidence genes, the thresholds of |log_2_FC| > 1 ∧ adj. *p* < 0.05 are used. The setting of the TCGA threshold was stricter (|log_2_FC| > 1 and adjusted *p* < 0.05) mainly because of its substantially larger sample size and lower data noise compared with the integrated GEO training set. To avoid losing important biological information in the smaller combined group, a less strict threshold was used in the GEO training set (FDR < 0.05, |log_2_FC| > 0.585). The following WGCNA and PPI analyses then offered extra filtering steps. This approach reduced the chance that the differential thresholding would significantly affect the selection of downstream candidates [22].

### 2.4. Weighted Gene Co-Expression Network Analysis (WGCNA) of DEGs

WGCNA (v1.73) was used to construct the co-expression network. The soft-threshold power β was selected based on the scale-free topology criterion: candidate β values from 1 to 20 were evaluated, and β = 6 was chosen as the minimum value at which the scale-free fitting index exceeded 0.90 while maintaining reasonable mean connectivity. This setting is consistent with the standard biological assumption of approximate scale-free topology in gene co-expression networks. Hierarchical clustering was performed based on the topology overlap matrix (TOM), and gene modules were identified by dynamic tree cutting (minimum module size = 50), and modules with a merge cut height < 0.25 were merged [23]. Through the correlation analysis of module feature genes and clinical phenotypes, we screened the modules that were significantly correlated with BC (|correlation coefficient| > 0.6, *p* < 0.001) and extracted the genes with high gene significance (|GS| > 0.4) and high intra-module connectivity (|MM| > 0.8) as genes, which were further intersected with the DEGs of GEO and TCGA to take the intersections for subsequent Protein–Protein Interaction Network (PPI) Analysis [24,25].

### 2.5. PPI Analysis of DEGs

The protein interaction network was constructed based on the STRING database (v11, interaction score threshold ≥ 400), and the comprehensive score of each gene was calculated by global centrality analysis (five indexes: Betweenness, Closeness, Degree, Eigenvector centrality, and PageRank) and then normalized and weighted by Z-score with the following weights: Betweenness (0.25), Closeness (0.25), Degree (0.20), Eigenvector centrality (0.15), and PageRank (0.15). Higher weights were assigned to Betweenness and Closeness because these measures better capture the bottleneck and global accessibility characteristics of network hubs; the genes with the top 30 comprehensive scores were screened as candidate key sets for ML model construction [26]. The network topology parameters (density, average path length, clustering coefficient) were further calculated, and centrality distribution maps, gene heat maps, PPI network maps, and high-intensity interactions and chordal maps were drawn.

#### Feature Selection and Hub Gene Identification Based on Integrated ML and SHAP Algorithm

Candidate genes obtained through PPI network analysis were incorporated into a multi-model ML framework containing 127 algorithms covering regularized models, generalized linear models, integrated learning methods, pattern recognition models, and multi-class gradient boosting methods for extensive model performance exploration [27]. The modeling process is based on Recursive Feature Elimination (RFE) for variable screening and stacked generalization to construct a multi-model prediction system [28]. All candidate models were optimized and fitted within a hierarchical 10-fold cross-validation framework. To reduce the overfitting risk associated with evaluating 127 model combinations on a limited training set, we applied recursive feature elimination before model fitting and further assessed model generalizability in four independent external validation cohorts. Nevertheless, we acknowledge that some degree of model selection bias cannot be fully excluded given the ratio between sample size and model space, and this limitation has now been explicitly noted. SHAP (SHapley Additive exPlanations) was used to quantify the feature contributions, the kernelshap (version 0.9.1) and shapviz (version 0.10.3) packages were combined to realize the model interpretability analysis, and the custom optimized prediction function was used to perform bootstrap resampling (n = 100) to calculate the feature average SHAP values and their 95% confidence intervals [29]. Hub genes were extracted based on the best-performing models, and their stability was verified in the training set and external validation set. Optimal hub genes were filtered based on the area under the ROC curve (AUC).

### 2.6. Functional Annotation and Gene Interaction Network Analysis

Functional enrichment and gene interaction network analyses were performed on hub genes identified through external validation screening. The R packages “clusterProfiler” and “DOSE” (version 4.4.0) were used for GO functional annotation and KEGG pathway enrichment analysis to investigate their potential roles in biological processes, molecular functions, cellular components, and signaling pathways in BC [30]. To further analyze their gene interaction network in BC, a gene interaction network was predicted and constructed using the GeneMANIA database (www.genemania.org; access date: 14 December 2025) [31], and the resulting network was visualized to identify functionally related genes and their interactions with hub genes.

### 2.7. Immune Infiltration Assessment and microRNA Regulation Analysis

The CIBERSORT algorithm was used to deconvolve the BC expression matrix based on linear support vector regression (https://cibersortx.stanford.edu/, accessed on 10 January 2026) to obtain the relative abundance information of 22 immune cell types covering multiple innate and adaptive immune cell populations and to evaluate [32]. The association patterns between hub genes and key immune cell types were evaluated, and the candidate microRNAs interacting with hub genes were predicted by the miRDB database (http://www.mirdb.org/, accessed on 11 January 2026) [33]. miRNA-mRNA regulatory networks were constructed to reveal the key post-transcriptional regulatory axes for BC progression.

### 2.8. SMR-Based Causal Inference Analysis of Gene Expression–BC Risk

To validate the causal association of hub genes with BC risk at the genetic level, the data were further aggregated using the FinnGen genome-wide association study (GWAS) and standardized and preprocessed to generate the SMR input format for Mendelian randomization analysis based on expression quantitative trait loci in combination with eQTLGen cis-eQTL data [34]. The analysis was performed using a dual screening framework: an FDR-corrected threshold (*p*_SMR < 0.05) was used to identify significant signals, and was then combined with the HEIDI test (*p*_HEIDI > 0.05) to exclude false positives due to linkage disequilibrium, thus obtaining candidate genes that supported causal variants shared with BC risk [35]. SMR region maps with effect scatter plots were drawn for hub genes, and Manhattan plots were generated based on genome-wide results to demonstrate the causal structure between expression regulation and disease risk.

### 2.9. Single-Cell Spatial Transcriptome Data Analysis and Hub Gene Expression Validation

This study was based on the BC single-cell RNA sequencing dataset GSE186344, which was systematically analyzed using Seurat (version 5.3.1). The raw UMI matrix was quality-controlled to retain high-quality cells with >200 genes and <15% mitochondrial genes and normalized using the LogNormalize method. Subsequently, PCA downscaling was performed, and Harmony was used to correct for batch effects between samples [36]. A k-nearest neighbor graph was constructed based on the first 20 principal components, and clustering was performed at a resolution of 0.6. Cell type annotation was performed using SingleR (version 2.12.0) in conjunction with the Human Primary Cell Atlas reference dataset [37]. The expression characteristics of hub genes in different cell subpopulations can be visualized through FeaturePlot, VlnPlot, and DotPlot, and their expression differences between tumor and normal tissues can be compared.

### 2.10. Drug Repurposing Analysis and Compound Prioritization

Drug–gene association screening was performed using DSigDB through the clusterProfiler package (version 4.18.2) with significance thresholds of *p* < 0.05 and FDR < 0.05, identifying small molecules associated with the hub gene expression patterns [38].

Automated druggability assessment was conducted using a multidimensional scoring framework. The system integrates seven established drug-likeness criteria: (1) Lipinski’s rule of five, (2) Veber’s rule, (3) Ghose’s rule, (4) quantitative estimate of drug-likeness (QED), (5) synthetic accessibility score, (6) rule-based ADMET prediction, and (7) PAINS/BRENK structural alerts. A weighted linear model was employed:

(1) $$S_{\mathrm{total}}=\sum_{i=1}^{7}w_{i}\cdot S_{i}$$

with the specific weights

(2) $$S_{\mathrm{total}}=0.15\cdot S_{\mathrm{Lipinski}}+0.10\cdot S_{\mathrm{Veber}}+0.10\cdot S_{\mathrm{Ghose}}+0.20\cdot S_{\mathrm{QED}}+0.15\cdot S_{\mathrm{SA}}+0.20\cdot S_{\mathrm{ADMET}}+0.10\cdot S_{PAINS}$$

Each dimension score $S_{i}$ was normalized to the range [0, 1]. Weight assignments reflect the relative importance of each criterion in predicting in vivo drug success: QED (0.20) and ADMET (0.20) receive the highest weights as the two strongest predictors of clinical attrition; Lipinski (0.15) and SA score (0.15) are assigned intermediate weights reflecting their established role in oral bioavailability and synthetic feasibility; Veber (0.10), Ghose (0.10), and PAINS (0.10) receive lower weights as supplementary filters. This weighting scheme was informed by published analyses of clinical drug failure rates attributable to each property domain. The detailed scoring algorithm for each dimension can be found in Supplementary Table S1. Compounds were automatically classified into five grades:

(3) $$\mathrm{Grade}=\left\{\begin{array}{ll} A\;(\mathrm{Excellent}) & S_{\mathrm{total}}\geq 0.85\\ B\;(\mathrm{Good}) & 0.70\leq S_{\mathrm{total}}<0.85\\ C\;(\mathrm{Medium}) & 0.55\leq S_{\mathrm{total}}<0.70\\ D\;(\mathrm{Poor}) & 0.40\leq S_{\mathrm{total}}<0.55\\ F\;(\mathrm{Unqualified}) & S_{\mathrm{total}}<0.40 \end{array}\right.$$

Only A-grade compounds ($S_{\mathrm{total}}\geq 0.85$) were selected for subsequent molecular docking analysis.

### 2.11. Molecular Docking and Protein–Ligand Interaction Analysis

Automated high-throughput molecular docking was performed using Smina (Vina scoring function) for Grade A candidate compounds. Protein structures were obtained from RCSB PDB, with screening criteria including Homo sapiens, X-ray resolution < 3.0 Å, single-chain structure, and co-crystallized ligands with molecular weights ≥ 100 Da. All structures were automatically protonated at pH 7.4 and converted to PDBQT format using OpenBabel (Version 3.1.1) [39].

Automatic docking box generation is based on eutectic ligands. The geometric centers and sizes of the ligand atomic coordinates are calculated by the following equations:

(4) $$\mathrm{Center}=\frac{1}{2}\left[max\left(x_{i},y_{i},z_{i}\right)+min\left(x_{i},y_{i},z_{i}\right)\right]$$

(5) $$\mathrm{Size}=max\left(x_{i}\right)-min\left(x_{i}\right)+2\delta ,\;max\left(y_{i}\right)-min\left(y_{i}\right)+2\delta ,\;max\left(z_{i}\right)-min\left(z_{i}\right)+2\delta$$

where δ=4.0Å is the padding distance. All docking tasks were run in an automated parallel optimization environment, with exhaustiveness set to 16.

Conformational stability validation was performed by automated RMSD redocking (threshold: 2.0 Å). The RMSD between the highest-scoring conformation and the redocked conformation was calculated as

(6) $$\mathrm{RMSD}=\sqrt{\frac{1}{N}\sum_{i=1}^{N}{\left|r_{i}-r_{\prime }^{i}\right|}^{2}}$$

where $r_{i}$ and $r_{\prime }^{i}$ are the atomic coordinates of the original and redocked conformations, respectively.

Interaction analysis was performed according to PLIP standards, identifying hydrogen bonds (≤3.5 Å), salt bridges (≤5.5 Å), hydrophobic interactions (≤4.0 Å), and π interactions (≤5.5 Å) [40]. Three-dimensional visualization was achieved through the PyMOL (Version 3.0.3) API to analyze key binding modes of candidate complexes.

## 3. Results

### 3.1. DEGs Analysis of BC Based on GEO and TCGA Data

**Figure 2:**
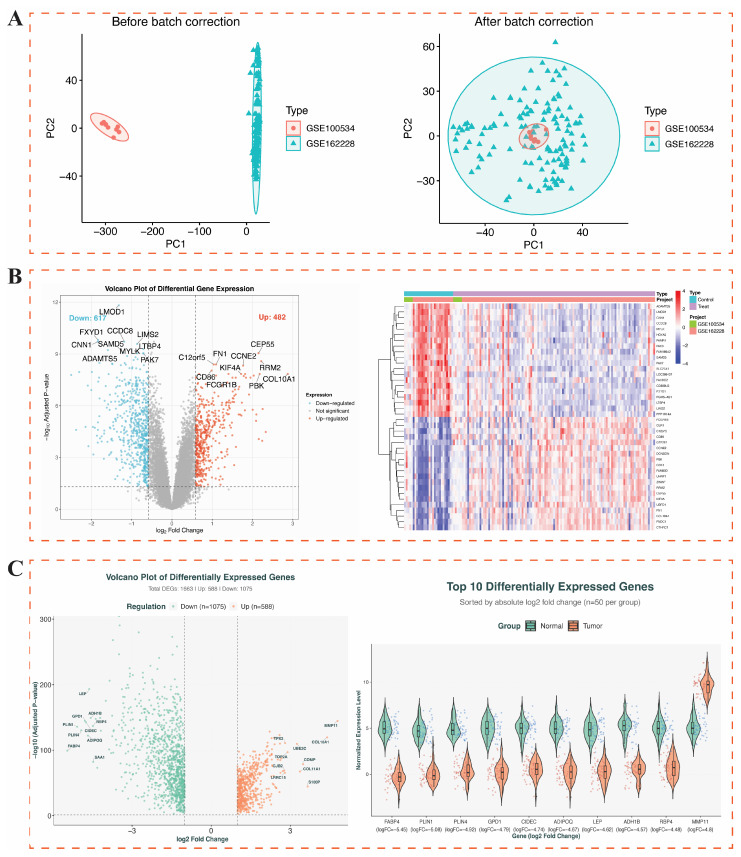
GEO and TCGA Data preprocessing. (**A**) Before batch calibration and after batch calibration of GSE100534 and GSE162228. (**B**) Screening of DEGs in GEO volcano plot and heatmap. (**C**) Screening of DEGs in TCGA volcano plot and violin plot.

Two datasets (GSE100534, GSE162228) were selected for model training in this study. Preliminary analysis showed that there were significant batch differences in the datasets. To effectively integrate the data, principal component analysis (PCA) was used to process the data, which successfully eliminated the batch differences (Figure 2A). On this basis, differential expression analysis was performed using the limma software package with the screening criteria of FDR < 0.05 and |Log_2_FC| > 0.585, and a total of 1099 differentially expressed genes (DEGs), including 482 up-regulated genes and 617 down-regulated genes, were identified (Figure 2B). To further improve the reliability of the results, this study also combined the clinicopathologic data of 1231 BC from the TCGA database with more stringent screening criteria (|log_2_FC| > 1 and corrected *p* value < 0.05), from which 588 up-regulated genes and 1073 down-regulated genes were identified (Figure 2C). These identified DEGs from both the GEO training set and TCGA dataset were subsequently incorporated into the weighted gene co-expression network analysis (WGCNA) to identify BC-associated gene modules.

### 3.2. WGCNA and Screening of Potential BC-Related Genes

**Figure 3:**
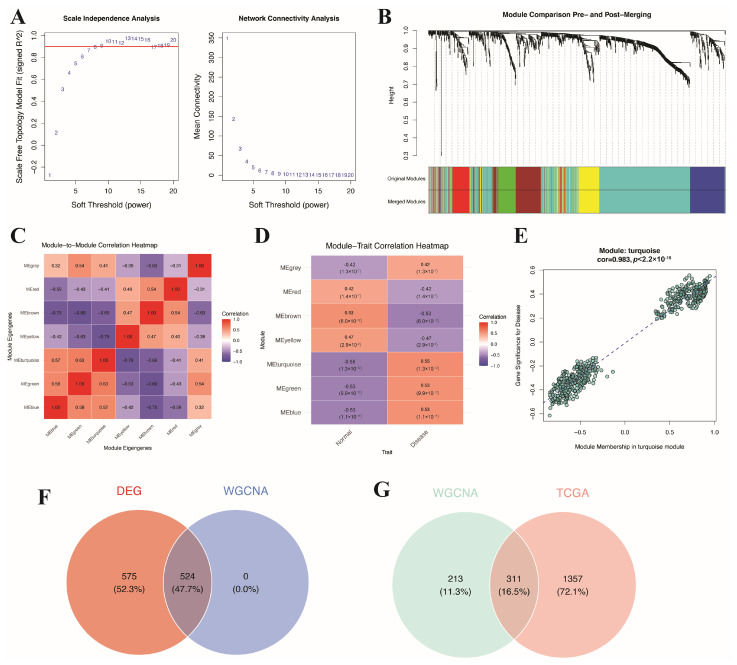
WGCNA. (**A**) Network topology analysis under different soft threshold power (ß): (**Left**) scale-free fitting index. The red horizontal line indicates the threshold for scale-free topology fit index (R^2^ = 0.90); (**Right**) average connectivity. (**B**) Hierarchical clustering dendrogram of genes and module division, with different colors representing different modules. (**C**) Correlation heatmap between modules. (**D**) Correlation heatmap between module characteristic genes and BC expression levels. (**E**) Module characteristics most significantly associated with BC. (**F**) Intersection of WGCNA module genes and GEO dataset DEGs. (**G**) Intersection of the genes from Figure 3F and TCGA dataset DEGs.

After data integration and the screening of DEGs, further analysis was conducted using WGCNA to identify gene modules associated with BC. The optimal soft threshold was determined by calculating the scale-free index and average connectivity at different soft threshold powers (β), thereby constructing a gene network (Figure 3A,B). Based on hierarchical clustering and the dynamic tree cutting method, multiple gene modules were successfully identified, among which the MEturquoise module was significantly associated with the expression levels of BC, containing 524 genes (Figure 3C–E). Since the WGCNA was constructed using the filtered DEGs as input, all genes in the identified key module were DEGs by design. Subsequently, by combining DEGs from the GEO dataset, an intersection analysis with the genes in the MEturquoise module identified 524 potential related genes (Figure 3F). Further intersection of these genes with DEGs in the TCGA dataset ultimately yielded 311 intersecting genes (Figure 3G). These 311 intersecting genes were subsequently subjected to PPI network analysis to identify hub genes with high network centrality.

### 3.3. PPI Analysis

**Figure 4:**
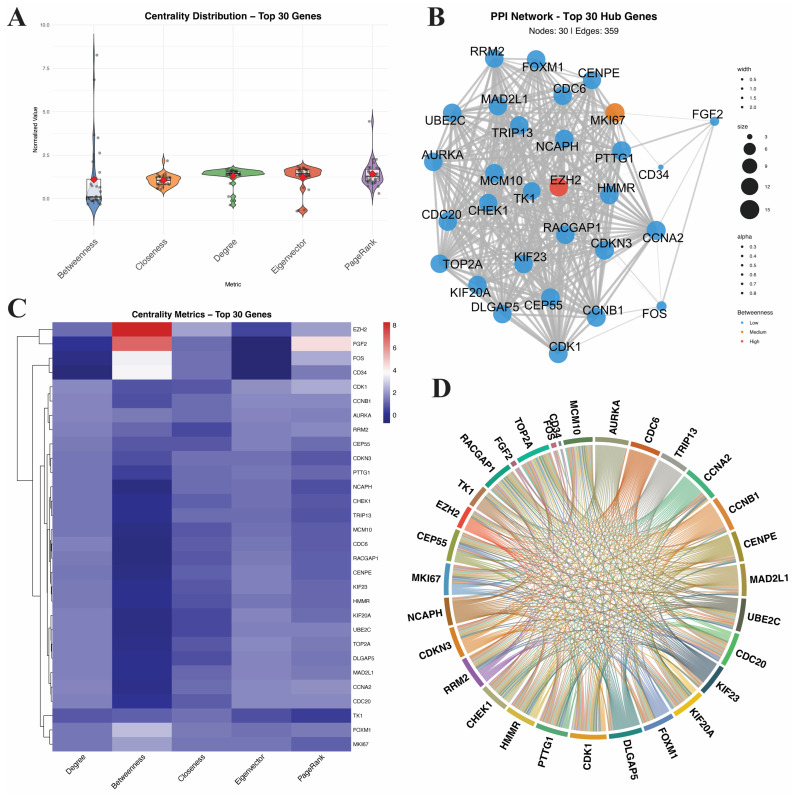
PPI Analysis. (**A**) Centrality index distribution. (**B**) PPI network structure. (**C**) Centrality heatmap analysis. (**D**) Gene interaction relationship diagram.

In this study, a PPI network was constructed using the 311 intersecting genes derived from WGCNA and DEG analyses to identify key genes in BC. Figure 4A illustrates the distribution characteristics of the top 30 genes using the five standardized centrality metrics. The violin plot shows that Betweenness exhibits the greatest variability, with multiple high-value outliers (the highest normalized value reaches about 8.26). In contrast, Closeness, Degree, Eigenvector, and PageRank all show relatively centralized distribution patterns with low inter-gene variability. Network visualization (Figure 4B) further revealed that genes such as CHEK1, KIF23, and EZH2 were at the core of interactions with the highest connectivity. Heat map analysis (Figure 4C) clustered genes based on multi-dimensional centrality, in which genes such as EZH2 and FGF2 were prominent in multiple centrality indicators. The chord diagram (Figure 4D) delineated the interaction strengths and modular relationships among the key genes. Based on these identified key genes, ML models were subsequently constructed to evaluate their predictive performance in BC classification.

### 3.4. Constructing a Diagnostic Model of BC by ML

**Figure 5:**
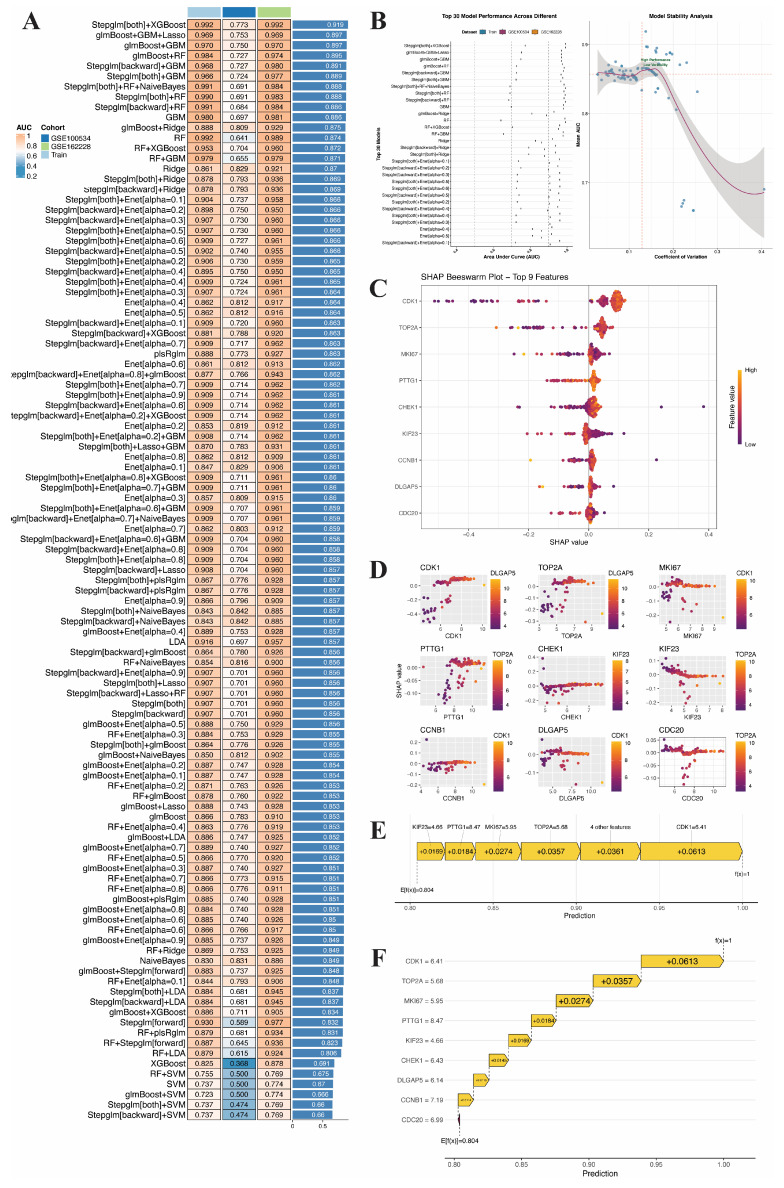
ML Model construction, selection, and SHAP interpretability analysis. (**A**) Heatmap of the performance of 127 ML models. (**B**) Model stability analysis. Vertical and horizontal dashed lines represent the median coefficient of variation and AUC across models, respectively. (**C**) SHAP summary bee swarm plot. (**D**) SHAP dependence plots. (**E**) Global SHAP waterfall plot. (**F**) Single-sample SHAP waterfall plot.

Based on the 30 candidate genes screened from the PPI network, 127 ML algorithms combined with various feature selection strategies were used to construct 127 “feature selection-classifier” combinations and systematically evaluate their diagnostic performance. The heatmap comparison (Figure 5A) shows that “StepGlm[both] + XGBoost” achieved the highest average AUC (0.919) in the training set. Stability analysis (Figure 5B) indicates that this combination has both a high AUC and a low coefficient of variation, making its overall performance and consistency the best; thus, it was determined to be the final diagnostic model.

The optimal model was subjected to SHAP global interpretation, and the bee swarm plot (Figure 5C) identified nine core feature genes: CDK1, TOP2A, MKI67, PTTG1, CHEK1, KIF23, CCNB1, DLGAP5, and CDC20. The dependency graph (Figure 5D) shows a nonlinear relationship and interaction effects between gene expression and contribution. The population-level waterfall plot (Figure 5E) quantifies the cumulative contributions of features, with CDK1 contributing the most (+0.0613), followed by TOP2A (+0.0357) and MKI67 (+0.0274); the single-sample waterfall plot (Figure 5F) reproduces this contribution ranking at the individual level. To further validate the reliability of the aforementioned nine core feature genes, external independent datasets were subsequently introduced for systematic validation.

### 3.5. External Validation of SHAP-Identified Hub Genes

**Figure 6:**
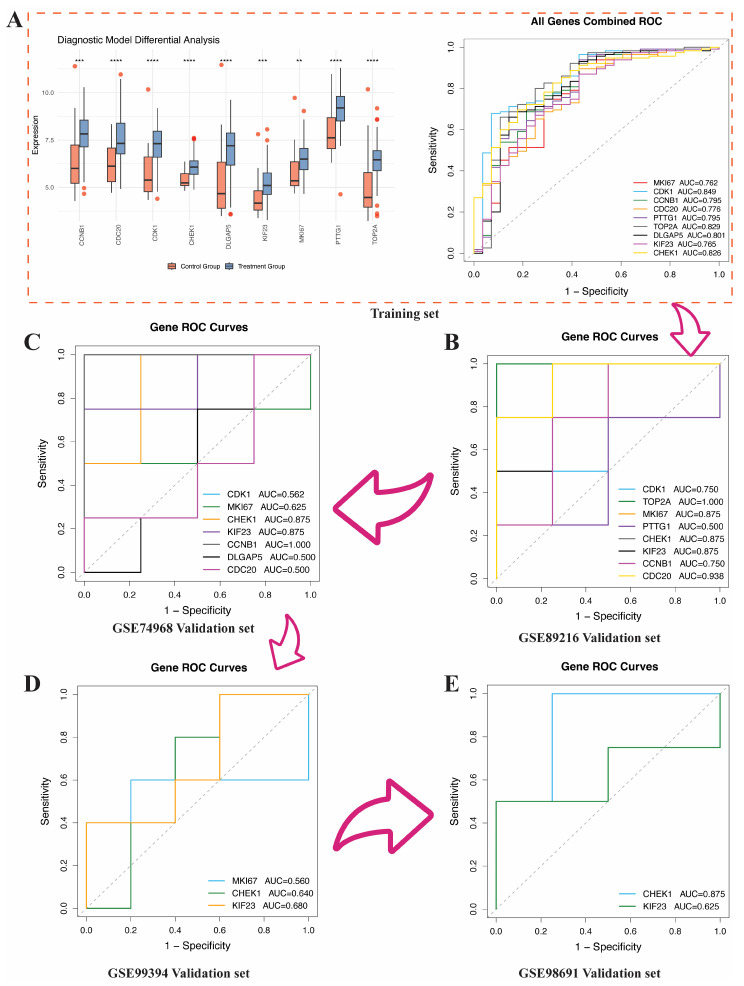
Identification and external validation of diagnostic genes. (**A**) Differential expression. Asterisks indicate statistical significance (** *p* < 0.01; *** *p* < 0.001; **** *p* < 0.0001) and ROC analysis in the training set. (**B**) ROC analysis in the GSE89216 validation set. (**C**) ROC analysis in the GSE74968 validation set. (**D**) ROC analysis in the GSE99394 validation set. (**E**) ROC analysis in the GSE98691 validation set.

To verify the stability and diagnostic value of the nine hub genes selected through SHAP analysis, this study evaluated their expression differences and diagnostic performance in four independent external validation cohorts. Figure 6A shows that in the training set, all candidate genes exhibited significant differential expression between the two groups, and ROC curve analysis indicated a certain disease discrimination ability, with AUC values ranging from 0.762 to 0.849. Figure 6B–E further present the ROC validation results of these genes in the four independent external datasets. To avoid overfitting and enhance the reliability of the results, this study excluded results with AUC = 1.000 and filtered out genes with AUC < 0.6.

In the GSE89216 dataset (Figure 6B), the AUCs of CDC20 and CHEK1 are 0.938 (95% CI: 0.750–1.000) and 0.875 (95% CI: 0.625–1.000), respectively. All AUC confidence intervals were calculated using bootstrap resampling (see Supplementary Table S2). In the GSE74968 dataset (Figure 6C), CHEK1 and KIF23 maintained good discrimination ability, both with an AUC of 0.875. In the GSE99394 dataset (Figure 6D), the AUCs for CHEK1 and KIF23 were 0.640 and 0.680, respectively. Similarly, in the GSE98691 dataset (Figure 6E), CHEK1 and KIF23 still exhibited certain diagnostic capabilities, with AUCs of 0.875 and 0.625, respectively.

### 3.6. Functional Characterization and Interaction Network Analysis of CHEK1 and KIF23

**Figure 7:**
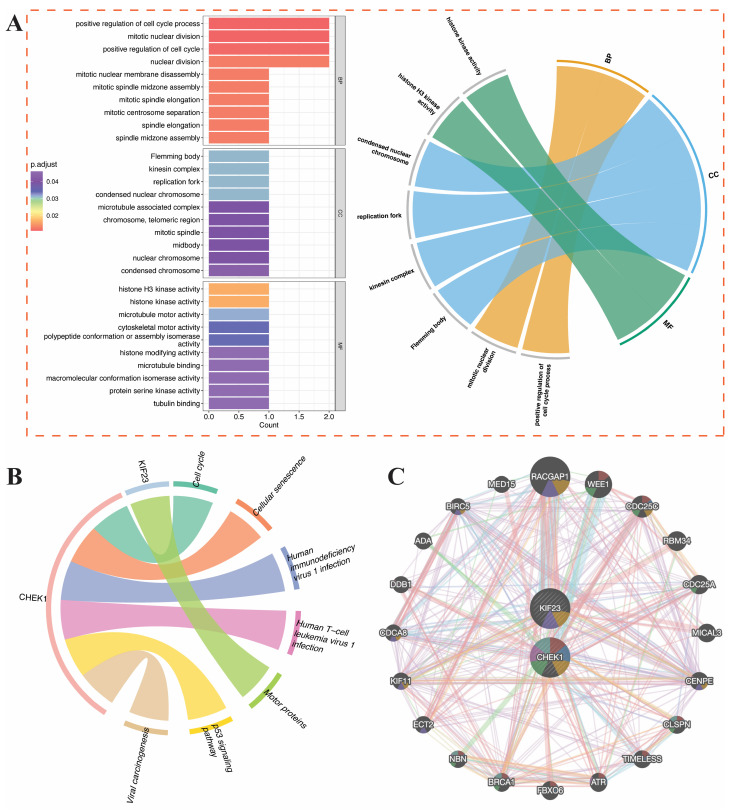
GO and KEGG enrichment analyses and gene interaction network of hub genes CHEK1 and KIF23 based on GeneMANIA. (**A**) GO enrichment analysis of differentially expressed genes across BP, CC, and MF categories. (**B**) Functional pathway enrichment analysis of key genes. (**C**) PPI network highlighting interactions among hub genes.

To clarify the biological functions and molecular interaction mechanisms of CHEK1 and KIF23, this study conducted GO enrichment analysis, KEGG pathway enrichment analysis, and gene interaction network construction. The GO enrichment analysis (Figure 7A) showed that the hub gene is mainly enriched in proliferation-related terms at the BP level, such as positive regulation of the cell cycle, mitotic nuclear division, and nuclear division. At the CC level, it is enriched in the Fleming body, kinesin complex, and replication fork, and at the MF level, it is mainly related to histone H3 kinase activity and microtubule motor activity. According to KEGG enrichment analysis, CHEK1 and KIF23 are significantly enriched in the cell cycle, cellular senescence, p53 signaling pathway, and viral carcinogenesis pathways (Figure 7B). CHEK1 and KIF23 were found to be associated with key genes such as BRCA1 and ATR through co-expression, physical interactions, and pathway sharing (Figure 7C). Next, we will investigate the potential roles of CHEK1 and KIF23 in the breast cancer immune system, as well as their roles in upstream miRNA regulation.

### 3.7. Immune Infiltration Analysis and miRNA Regulatory Network Construction of CHEK1 and KIF23

**Figure 8:**
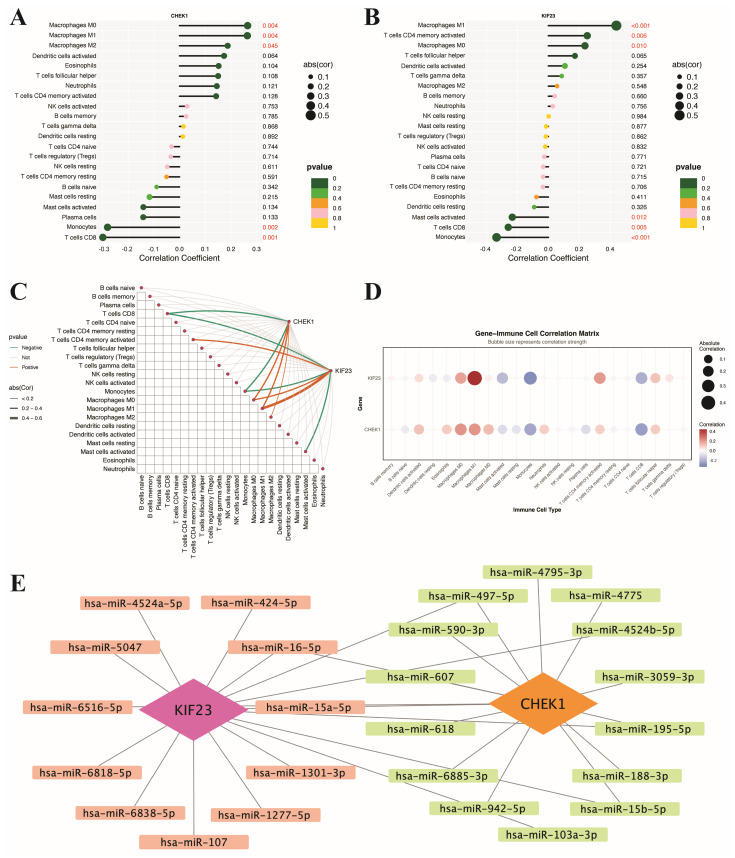
Immune infiltration correlation analysis and miRNA regulatory network construction of hub genes CHEK1 and KIF23. (**A**) Correlation between CHEK1 expression and immune cell infiltration. (**B**) Correlation between KIF23 expression and immune cell infiltration. (**C**) Integrated correlation network between hub genes and immune cells. (**D**) Gene–immune cell correlation matrix of CHEK1 and KIF23. (**E**) Predicted miRNA regulatory network of CHEK1 and KIF23.

To evaluate the potential roles of the hub genes CHEK1 and KIF23 in the immune microenvironment of BC and their upstream regulatory mechanisms, we analyzed the correlations between these two genes and 22 types of tumor-infiltrating immune cells using the CIBERSORT algorithm and constructed a predicted miRNA regulatory network. Immune infiltration analysis showed that CHEK1 expression was significantly positively correlated with macrophages M0, M1, and M2, while it was significantly negatively correlated with monocytes and CD8 T cells (Figure 8A). KIF23 expression was significantly positively correlated with macrophages M0, M1, and activated memory CD4 T cells, and significantly negatively correlated with activated mast cells, CD8 T cells, and monocytes (Figure 8B). The integrated correlation network between hub genes and immune cells (Figure 8C) and the gene–immune cell correlation matrix (Figure 8D) suggested relatively stronger correlations of KIF23 with monocytes and macrophages M1 compared with CHEK1. The predicted miRNA regulatory network analysis (Figure 8E) suggested that KIF23 may be regulated by miRNAs such as hsa-miR-16-5p, hsa-miR-15a-5p, and hsa-miR-107, whereas CHEK1 may be regulated by miRNAs such as hsa-miR-195-5p, hsa-miR-497-5p, and hsa-miR-15b-5p. hsa-miR-15a-5p and hsa-miR-607 are predicted to target both CHEK1 and KIF23, suggesting a shared upstream miRNA regulatory mechanism. To further assess the causal relationship between the expression of these genes and genetic susceptibility to breast cancer, a Mendelian randomization analysis based on SMR was subsequently conducted.

### 3.8. SMR-Based Mendelian Randomization Analysis of Hub Genes and BC Risk

**Figure 9:**
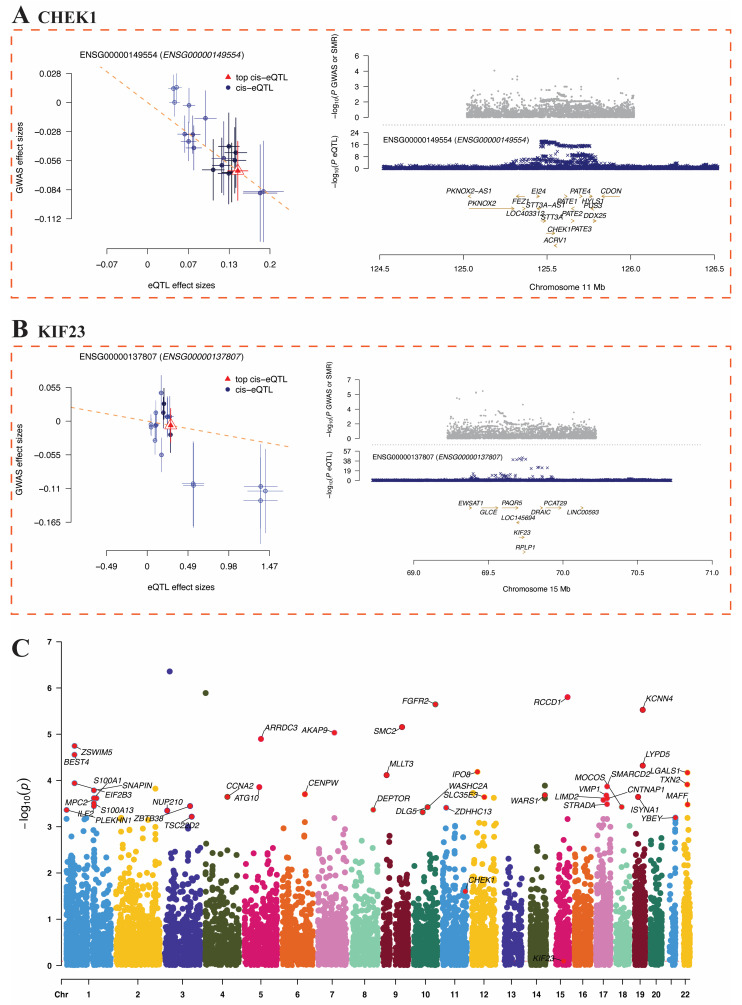
SMR analysis and GWAS locus visualization of hub genes CHEK1 and KIF23. (**A**) CHEK1: SMR scatter plot and co-localization in chromosome 11 region. Blue dots represent cis-eQTL signals, red triangles indicate the top cis-eQTL, arrows denote gene orientation, and different colors represent chromosomes. (**B**) KIF23: SMR scatter plot and co-localization in chromosome 15 region. (**C**) BC GWAS Manhattan plot (significant loci for CHEK1 marked in blue).

To explore the potential causal relationship between the expression levels of hub genes (CHEK1 and KIF23) and genetic susceptibility to BC, this study employed the SMR method to integrate cis-eQTL data with BC GWAS data for joint analysis. Figure 9A shows that the SMR analysis results for CHEK1 (ENSG00000149554) indicate a negative correlation trend between its top cis-eQTL and GWAS effect sizes; the colocalization plot for the chromosome 11q (124.5–126.5 Mb) region shows a significant distribution of eQTL signals in this area. The HEIDI test confirmed the absence of linkage disequilibrium confounding for CHEK1 (*p*_HEIDI > 0.05), supporting a shared causal variant underlying both gene expression and BC risk. Figure 9B shows that the SMR scatter plot for KIF23 (ENSG00000137807) also presents a negative correlation trend between eQTL and GWAS effects, with concentrated eQTL signals in the chromosome 15q (69.0–71.0 Mb) region, although it did not reach the SMR significance threshold. The further genome-wide SMR Manhattan plot (Figure 9C) shows that only CHEK1 in the chromosome 11q region exceeds the SMR significance threshold, while KIF23 did not receive significant support.

### 3.9. Single-Cell RNA Sequencing Validation of Hub Gene CHEK1 Expression

**Figure 10:**
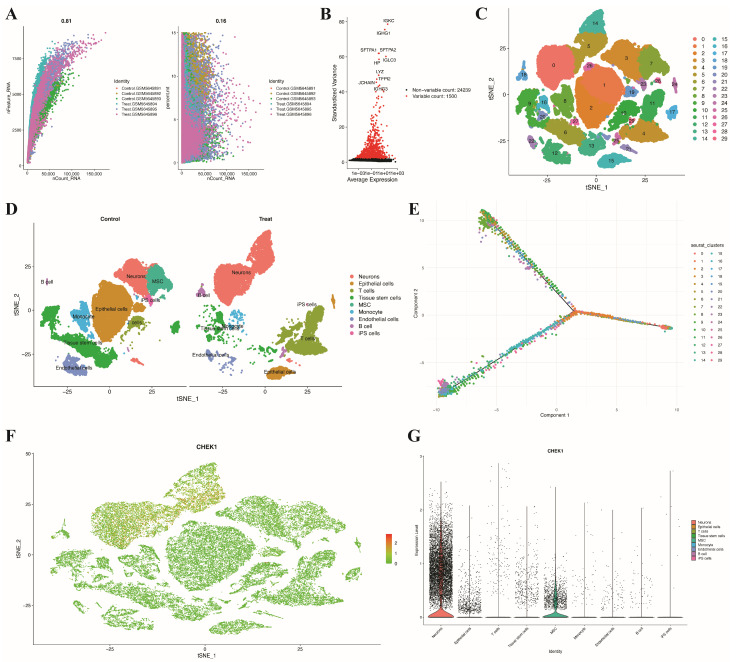
Single-cell RNA sequencing analysis reveals cellular heterogeneity and the expression pattern of hub gene CHEK1 in BC. (**A**) Quality control metrics of single-cell RNA sequencing data. (**B**) Identification of highly variable genes across single cells. (**C**) t-SNE visualization showing clustering of single cells. (**D**) Cell type annotation of clusters in control and treatment groups. (**E**) Trajectory analysis of cellular differentiation states. (**F**) t-SNE visualization of CHEK1 expression distribution across single cells. (**G**) Violin plot showing CHEK1 expression levels across different cell types.

To further validate the expression characteristics of hub genes at the single-cell level, this study analyzed single-cell RNA sequencing data (GSE186344) of BC (Figure 10). Figure 10A shows the quality control results of the single-cell data, including the distribution of nCount_RNA and nFeature_RNA, as well as the proportion of mitochondrial genes (percent.mt). A total of 1500 highly variable genes were identified through high-variance gene screening (Figure 10B), and all cells were clustered into 30 subpopulations using t-SNE dimensionality reduction (Figure 10C). The cell type annotation results (Figure 10D) indicate that both the control group and the treatment group contain major cell types such as epithelial cells, T cells, B cells, monocytes, endothelial cells, and mesenchymal cells, but there are differences in cell composition between the two groups. Pseudotime trajectory analysis (Figure 10E) revealed the differentiation trajectories among the cell populations. CHEK1 expression exhibited heterogeneous patterns at the single-cell level (Figure 10F). The violin plot shows that CHEK1 is highly expressed in neuronal cells but lowly expressed in other cell types (Figure 10G).

### 3.10. Drug Enrichment and Virtual Screening Analysis of CHEK1

**Figure 11:**
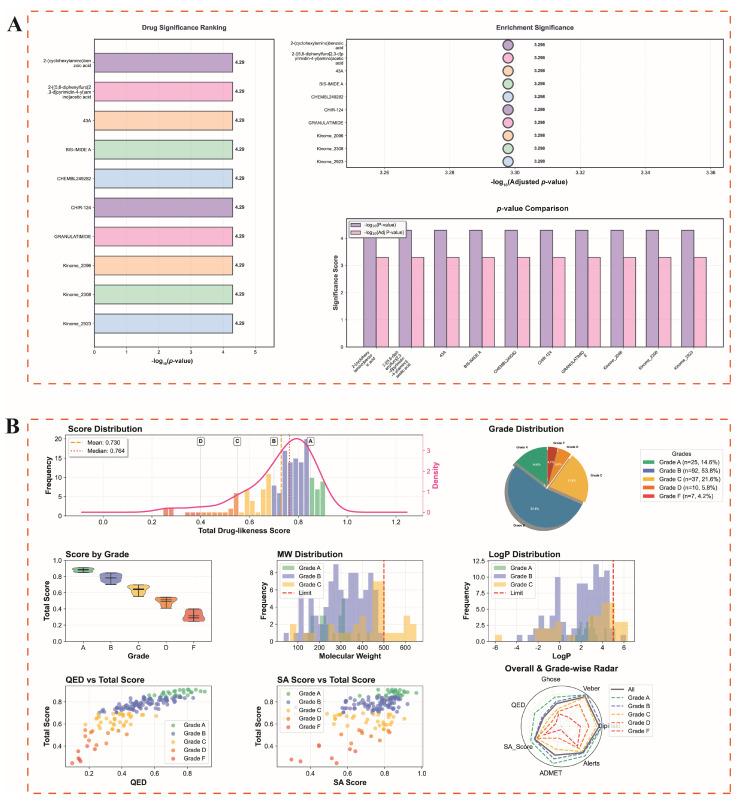
Drug enrichment analysis and virtual screening identify candidate compounds targeting CHEK1. (**A**) Significant analysis of hub gene-targeted drug enrichment. (**B**) Comprehensive evaluation of virtual screening of candidate compounds for drug-like properties. Colors represent different grade groups (A–F). Letters (A–D) indicate representative score ranges. Red dashed lines denote cutoff values. The radar plot summarizes compliance with Lipinski, Veber, and Ghose rules.

To screen candidate drugs targeting CHEK1, this study combined gene–drug enrichment analysis (Figure 11A) with artificial intelligence virtual screening (Figure 11B). The enrichment analysis identified a total of 188 significantly related drugs (*p*.adjust < 0.05), including 2-(cyclohexylamino) benzoic acid, BIS-IMIDE A, CHIR-124, GRANULATIMIDE, CHEMBL249282, and Kinome series compounds (2098, 2308, 2923), among others. After removing duplicate entries and compounds lacking valid structural information (e.g., unavailable SMILES), 171 compounds were retained for subsequent virtual screening.

The drug-likeness virtual screening (Figure 11B) evaluated these 171 candidate compounds, showing an average drug similarity score of 0.730 and a median of 0.764. By ranking, Grade B accounted for the highest proportion (53.8%, *n =* 92), followed by Grade C (21.6%, *n* = 37), Grade A (14.6%, *n* = 25), Grade D (5.8%, *n* = 10), and Grade F (4.1%, *n* = 7). The distribution analysis of molecular weight (MW) and lipophilicity (LogP) indicated that both Grade A and Grade B compounds met the five principles of Lipinski’s rule of five. The scatter plot of QED and SA scores showed that Grade A compounds concentrated in the high QED (0.4–0.8) and high synthetic accessibility (SA score 0.6–0.8) regions, with overall scores generally above 0.7. Radar chart analysis further indicated that Grade A compounds had higher comprehensive scores than other grades across multiple drug-like property indicators such as Ghose, Veber, Lipinski, QED, and ADMET, and had lower structural alert levels; Grade B compounds followed, while Grade C–F compounds scored significantly lower on multiple indicators. Based on the above screening results, Grade A candidate compounds were further used for subsequent molecular docking analysis.

### 3.11. Molecular Docking Analysis of Candidate Compounds with CHEK1

**Figure 12:**
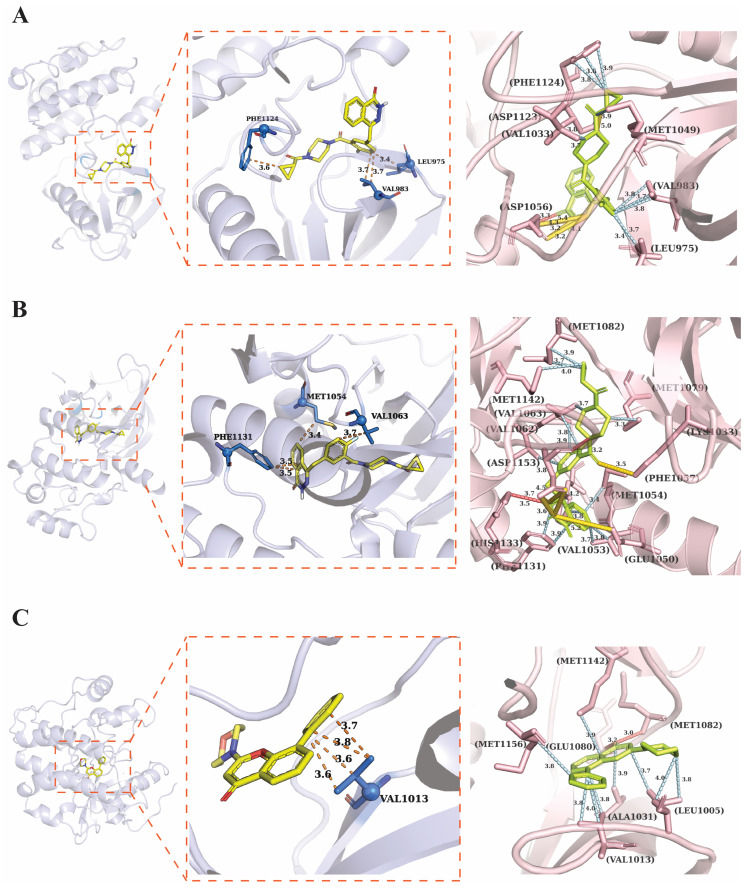
Molecular docking analysis showing the binding modes and ligand–protein interactions of candidate compounds with the CHEK1 kinase domain. (**A**) Binding conformation and interactions of Olaparib with CHEK1 (PDB: 3D94). (**B**) Docking interaction of Olaparib with CHEK1 (PDB: 3O23). (**C**) Binding mode and ligand–protein interaction network of LY294002 with CHEK1 (PDB: 5FXS).

To evaluate the binding mode of Grade A candidate compounds with CHEK1, molecular docking analysis was conducted (Figure 12 and Table 1). Figure 12A shows that the docking binding energy of Olaparib with the CHEK1 crystal structure (PDB: 3D94) is −10.2 kcal/mol. Its polar groups form hydrogen bond interactions with the main chain of VAL983 and LEU975 (bond lengths of 3.6 Å and 3.7 Å, respectively), while the hydrophobic backbone forms extensive hydrophobic contacts with residues such as PHE1124, ASP1123, VAL1033, MET1049, and ASP1056 (3.2–5.0 Å). Furthermore, the docking of Olaparib with CHEK1 (PDB: 3O23) (Figure 12B) shows a binding energy of −10.1 kcal/mol, where it forms multiple hydrogen bonds with PHE1131, MET1054, and VAL1063 in the binding pocket and has hydrophobic interactions with residues such as MET1082, MET1142, VAL1062, ASP1153, GLU1050, VAL1053, LYS1033, and PHE1057. The docking results of LY294002 with CHEK1 (PDB: 5FXS) (Figure 12C) show a binding energy of −9.8 kcal/mol, forming multiple hydrogen bonds with VAL1013 (3.6–3.8 Å), while also forming hydrophobic contacts with residues such as MET1156, GLU1080, MET1142, ALA1031, LEU1005, and MET1082 (3.0–4.0 Å).

## 4. Discussion

BC is the most common malignant tumor among women worldwide, posing a serious threat to women’s health. Advances in targeted therapy and immunotherapy have propelled improvements in diagnosis and treatment models, but the instability of early diagnostic markers and the lack of effective targeted drugs still limit the improvement of patient prognosis. Constructing precise molecular diagnostic models based on ML has significant clinical value in enhancing the early diagnosis of BC.

The ‘StepGlm[both] + XGBoost’ ensemble model (AUC = 0.919) constructed in this study demonstrated reproducible performance across four independent external cohorts. Compared with previous BC biomarker studies that typically relied on a single ML model or limited validation, our framework further incorporates multi-cohort integration, SHAP-based interpretation, SMR-based genetic support, and single-cell validation, thereby providing a more comprehensive perspective on CHEK1 as a candidate diagnostic and therapeutic target [41]. Compared with previously reported single-model or limited-model BC biomarker studies, our framework emphasizes broader model exploration and interpretability. However, we do not claim methodological superiority in the absence of direct head-to-head benchmarking against recent state-of-the-art approaches. CHEK1 and KIF23 demonstrated preliminary diagnostic potential in all validation cohorts (AUC range: 0.625–0.938; 95% confidence intervals estimated by bootstrap resampling). Given the small sample size of the external validation cohorts (4–5 cases per group), the robustness of the above AUC estimates requires support from larger independent cohorts, consistent with recent reports on CHEK1’s diagnostic value in triple-negative and hormone receptor-positive BC [42], providing cross-cohort evidence for its role as a core diagnostic biomarker. Based on these preliminary diagnostic findings, functional enrichment and interaction network analyses were subsequently performed to further characterize the potential biological roles of CHEK1 and KIF23.

CHEK1 and KIF23 are highly concentrated in the pathways of the cell cycle, p53 signaling, and cellular aging. CHEK1, as a core kinase of the ATR-CHEK1 axis, plays a key role in the DNA damage-induced G2/M checkpoint arrest. Overexpression of CHEK1 confers increased resistance to replication stress in tumor cells, thereby promoting tumor cell survival [43]. The other genes identified by SHAP, besides CHEK1 and KIF23, include CDK1, TOP2A, MKI67, PTTG1, CCNB1, DLGAP5, and CDC20. These genes are all closely related to the cell cycle, mitotic regulation, and signals that promote cell division. The fact that these genes are all enriched in cell-cycle and mitotic pathways supports the biological relevance of the diagnostic gene set that was found. GeneMANIA network analysis reveals that CHEK1, BRCA1, and ATR form a tightly coordinated regulatory network. This suggests that they may play a role in the development of BC through the homologous recombination repair pathway. This corresponds with data that designate CHEK1 as a synthetically lethal target in BRCA-deficient malignancies [44]. KIF23, as a mitotic driver protein, exhibits overexpression correlated with multiple tumor proliferations and poor prognosis [45], potentially acting as an independent proliferative driver synergistically involved in BC development.

Immune infiltration analysis demonstrated that CHEK1 expression was positively correlated with M0 and M1 macrophages and negatively correlated with CD8^+^ T cells. This suggests that CHEK1 may play a role in creating an immunosuppressive milieu. The DNA damage response system can affect the remodeling of the tumor immune milieu by controlling PD-L1 expression [46]. This study gives us a theoretical basis for the idea that combining CHEK1 inhibitors with immune checkpoint inhibitors could have synergistic effects. hsa-miR-15a-5p and hsa-miR-607 function as prevalent upstream regulatory miRNAs for CHEK1 and KIF23. hsa-miR-15a-5p has been shown to have a wide range of anticancer effects [47,48], which are very helpful for finding new miRNA-based treatments.

SMR analysis based on eQTLGen and FinnGen GWAS data [49] indicated that CHEK1 passed the HEIDI test, providing evidence consistent with a shared genetic basis between CHEK1 expression and BC susceptibility. These findings support CHEK1 as a genetically supported candidate for further functional investigation, although they do not establish definitive causality. In contrast, KIF23 did not reach the same level of SMR support and should therefore not be interpreted at the same level as CHEK1.

Single-cell transcriptome analysis indicated variable CHEK1 expression among cell populations, with a relative enrichment observed in a neuronal-like cluster. The biological implications of this expression pattern, however, are still under investigation. Consequently, future research that integrates pathway activity analysis and cell-state characterization within more extensive single-cell datasets will be crucial for elucidating its significance. Considering recent evidence that tumor innervation may influence BC progression [50], the observed enrichment of CHEK1 in neuronal-like clusters may reflect a possible role in tumor–neural interaction niches or neural-like transcriptional programs. However, given the limited size of the GSE186344 dataset (three normal and three tumor samples), this finding should be considered exploratory and hypothesis-generating, requiring validation in larger and better-annotated single-cell datasets.

Through DSigDB enrichment analysis and an AI-based multidimensional drug-like property scoring framework, 25 Grade A compounds were selected from 171 candidate compounds. Olaparib, an FDA-approved PARP1/2 inhibitor, targets homologous recombination repair-deficient tumors via a “synthetic lethality” mechanism [51,52]. Molecular docking showed that Olaparib had the strongest binding affinity with two forms of CHEK1 (−10.2/−10.1 kcal/mol). This study offers computational validation for the combination of PARP inhibitors with CHEK1-targeted therapy, as CHEK1 inhibition increases the cytotoxicity of PARP inhibitors and yields synergistic effects in BRCA-deficient malignancies [53]. LY294002 binds strongly to CHEK1 (−9.8 kcal/mol), which suggests that the PI3K/AKT pathway and the CHEK1 cell cycle checkpoint may be able to affect each other [54]. The three compounds under consideration exhibited promising in silico binding conformations within the CHEK1 active site, suggesting their potential as computationally identified CHEK1-targeting candidates for further investigation. Nevertheless, these docking results represent static structural predictions and therefore require additional experimental validation before any therapeutic implications can be inferred.

This investigation, relying on a retrospective public dataset, lacks experimental validation—specifically, techniques like qPCR, Western blotting, or functional assays—to substantiate the biological functions of CHEK1 or KIF23; the analysis of immune infiltration relied solely on computational deconvolution using CIBERSORT. This method estimates the relative proportions of immune cells based on reference gene signatures. However, it does not provide information about spatial organization or functional activation states. Therefore, these findings should be seen as preliminary and require experimental validation using methods like immunohistochemistry, flow cytometry, or spatial transcriptomics; molecular docking only reflects static binding conformations and cannot fully simulate dynamic protein–ligand interactions. Future work should combine prospective cohorts with in vitro and in vivo experiments.

## 5. Conclusions

This study identifies CHEK1 as a core gene in breast cancer through multi-omics integration, 127 ML algorithms, and genetic causal inference, and screens candidate compounds targeting CHEK1, providing potential experimental strategies for targeted therapy in BC.

## Figures and Tables

**Table 1:** Molecular docking information table.

Compound Name	Smiles	PDB	Pocket Center	Pocket Size	Affinity (kcal/mol)
Olaparib	C1CC1C(=O)N2CCN(CC2)C(=O)C3=C(C=CC(=C3)CC4=NNC(=O)C5=CC=CC=C54)F	3D94	[25.161499, 18.3265, −4.3955]	[10.0910015, 15.862999, 18.397]	−10.2
Olaparib	C1CC1C(=O)N2CCN(CC2)C(=O)C3=C(C=CC(=C3)CC4=NNC(=O)C5=CC=CC=C54)F	3O23	[7.4795, 1.2415001, 21.691]	[11.247, 18.867, 9.73]	−10.1
LY 294002	C1COCCN1C2=CC(=O)C3=C(O2)C(=CC=C3)C4=CC=CC=C4	5FXS	[8.952001, −4.277, 51.9955]	[19.462002, 10.378, 10.753002]	−9.8

## Data Availability

The datasets and materials used in this study are publicly available from the following sources: GEO datasets—GSE100534, GSE162228, GSE89216, GSE74968, GSE99394, GSE98691, GSE186344 (https://www.ncbi.nlm.nih.gov/geo/, accessed on 01 January 2026); TCGA-BC data—https://portal.gdc.cancer.gov/, accessed on 3 January 2026; PDB structures—3D94, 3O23, 5FXS (https://www.rcsb.org/, accessed on 6 February 2026). Compound SMILES: Olaparib: C1CC1C(=O)N2CCN(CC2)C(=O)C3=C(C=CC(=C3)CC4=NNC(=O)C5=CC=CC=C54)F LY294002: C1COCCN1C2=CC(=O)C3=C(O2)C(=CC=C3)C4=CC=CC=C4.

## Supplementary Materials

The following supporting information can be downloaded at https://www.mdpi.com/article/10.3390/genes17040396/s1. Table S1: Multidimensional Drug-Likeness Scoring Framework: Criteria, Algorithms, Weights, and Primary References; Table S2: AUC values of the training set and various validation sets.

## Abbreviations

The following abbreviations are used in this manuscript:

**Abbreviation**	**Full name**
BC	Breast Cancer
GEO	Gene Expression Omnibus
TCGA	The Cancer Genome Atlas
DEA	Differential Expression Analysis
DEGs	Differentially Expressed Genes
ML	Machine Learning
WGCNA	Weighted Gene Co-expression Network Analysis
PPI	Protein–Protein Interaction
SHAP	SHapley Additive exPlanations
PCA	Principal Component Analysis
FDR	False Discovery Rate
FC	Fold Change
ROC	Receiver Operating Characteristic
AUC	Area Under the Curve
GO	Gene Ontology
KEGG	Kyoto Encyclopedia of Genes and Genomes
SMR	Summary-based Mendelian Randomization
GWAS	Genome-Wide Association Study
eQTL	Expression Quantitative Trait Loci
HEIDI	Heterogeneity in Dependent Instruments
miRNA	MicroRNA
CIBERSORT	Cell-type Identification By Estimating Relative Subsets Of RNA Transcripts
DSigDB	Drug Signatures Database
ADMET	Absorption, Distribution, Metabolism, Excretion, and Toxicity
QED	Quantitative Estimate of Drug-likeness
RMSD	Root-Mean-Square Deviation
PLIP	Protein–Ligand Interaction Profiler
CHEK1	Checkpoint Kinase 1
KIF23	Kinesin Family Member 23
